# Substitutions in Fe_2_P Alloys for Permanent Magnet Applications

**DOI:** 10.3390/ma18051085

**Published:** 2025-02-28

**Authors:** Vasilios Panagopoulos, Athanasios Sigalos, Dimitrios I. Anyfantis, Dimitrios Niarchos

**Affiliations:** 1 Amen New Technologies, 15343 Athens, Greece; v.panagopoulos@inn.demokritos.gr (V.P.); a.sigalos@amen-tech.com (A.S.); 2 Institute of Nanoscience and Nanotechnology, National Centre for Scientific Research “Demokritos”, 15310 Athens, Greece

**Keywords:** Fe_2_P alloys, substitutions, magnetic properties, magnetization, coercivity, Curie temperature, permanent magnets

## Abstract

Fe_2_P (iron phosphide) alloys have garnered significant interest in recent years due to their potential applications in permanent magnet materials, particularly in the context of energy-efficient and environmentally friendly technologies. We have sought to tailor the magnetic properties, such as magnetization, coercivity, and Curie temperature, to meet the specific requirements of rare-earth-free permanent magnets for various industrial sectors. In this work, we review recent advancements in the exploration of substitutions (Si, Co, Mn, and Ni) within $Fe_{2}P$ alloys aimed at enhancing their magnetic performance as candidates for permanent magnets. The X-ray patterns of (Fe,Co)_2_P show great crystallinity with a pure Fe_2_P phase even with Mn and Ni substitutions. The Fe_2_P structure crystallizes in the P-62m space group. It has been confirmed that the transition metals substitute the 3g Fe-site, sometimes with adverse effects regarding magnetic properties with Co vs. Ni substitution, and that Si substitutes the 2c P-site. The saturation magnetization increases ($M_{S}=87\;Am^{2}/kg$) with Mn substitution, while the Curie temperature decreases with these substitutions. The impact of various substitutional elements on the magnetic properties of $Fe_{2}P$ alloys is highlighted, and challenges encountered in this field are reported.

## 1. Introduction

The demand for high-performance permanent magnets has surged in recent years, driven by advancements in technology and the increasing need for energy-efficient solutions across various applications, including electric motors, renewable energy systems, and consumer electronics [1]. Presently, two major magnet families are being manufactured at scale. The ferrite family, typified by phases such as $BaFe_{12}O_{19}$ and $SrFe_{12}O_{19}$, offers cost-effective and relatively straightforward production methods. However, their key metric, the energy product $|B{H|}_{max}$, is limited to approximately $44\;kJ/m^{3}$ [1]. Traditional permanent magnets, such as those based on rare-earth elements like neodymium (Nd) and dysprosium (Dy), provide exceptional magnetic properties but are often hampered by supply chain vulnerabilities and high costs [2]. These magnets can exhibit remarkable energy products reaching up to $480\;kJ/m^{3}$ [3]. Nd–Fe–B magnets are exceptionally high-performing, are primarily composed of iron, and can maintain suitable magnetic hysteresis at temperatures up to 200 °C by incorporating heavy rare earths such as Tb in place of Nd [4]. Nevertheless, the supply issues associated with heavy rare earths necessitate an exploration of alternatives to either replace or reduce the production costs and enhance the recycling of these magnets. Hence, the imperative to develop novel, cost-competitive materials devoid of rare earths, yet with an energy product bridging the gap between ferrite and rare-earth magnets, is paramount. Such materials could bolster performance and decrease the weight of devices currently reliant on ferrites or substantially cut down costs for those dependent on rare-earth magnets. Various families of 3d transition metal-based materials are under consideration as potential rare-earth-free permanent magnets, including MnBi [5], MnAl [6], $Mn_{2}Ga$ [7], $Fe_{16}N_{2}$ [8], $YCo_{5}$ [9], FePt and CoPt [10], and L1_0__FeNi [11]. Despite intensive research and promising properties having been achieved, no single preferred candidate has emerged definitively thus far.

Among the best candidates for rare-earth-free permanent magnets is the $Fe_{2}P$ family of compounds due to the abundance of their constituting elements and their significant magnetic properties. The Fe-rich family of the type $Fe_{2-x-y}Co_{x}{\left(Ni,Cr,Cu,Ti,V\right)}_{y}(P,Si)$ [12,13,14,15,16] is suitable for permanent magnet applications; conversely, the Mn–rich family $Mn_{2-x}{\left(Fe,V,Ti\right)}_{x}(P,Si)$ [17] is more attractive as a magnetocaloric material. The $Fe_{2}P$ compound undergoes a transition from a ferromagnetically ordered state to a paramagnetic state at 217 K [18], which is quite low for applications as a permanent magnet above room temperature or even for magnetocaloric applications (which require a Curie temperature, T_C_, around 300 K) [19]. By partially substituting Fe with other transition metals (e.g., Co, Ni, Cu, V, Ti, or Cr) and partially replacing P with Si, a wide tunability is achieved in crystal structure and magnetic properties, suitable for various applications [20]. However, these additions also lead to a more complex system; chemistry changes and extra phases emerge that can deteriorate the favorable properties of the hexagonal $Fe_{2}P$ phase. In the literature, there are more references to the Mn-rich family, suitable for magnetocaloric applications, regarding either the role of the substitution of Fe with other transition metals like W [21], Ta [22], Co [23], Ni [24], or Cr [25] or the replacement of P with Si [26,27,28]. On the other hand, very few reports exist on the role of substitutions involving transition elements or Si for the Fe-rich family of alloys [29,30], which can be considered candidates for permanent magnet applications. The main obstacle to achieving a permanent magnet based on modified $Fe_{2}P$ with acceptable properties as a gap magnet is not the magnetization or Curie temperature but rather the presence of a soft magnetic phase of the type $Fe_{3}(P,Si)$, which reduces the coercivity to 0.02–0.03 T [31] even at stoichiometries of 2–5% with respect to the tetragonal phase.

We have undertaken a systematic study to understand the influence of similar substitutions for the system $Fe_{2-x-y}Co_{x}{\left(Ni,Cr,Cu,Ti,V\right)}_{y}(P_{z-1},{Si}_{z})$, 0 < x < 1, 0 < y < 1 and 0 < z < 1. We present experimental data regarding their crystallographic evolution and provide a detailed magnetic study aimed at achieving a magnetic phase with optimal properties for rare-earth-free gap magnets.

## 2. Materials and Methods

The samples were synthesized through solid-state reactions employing stoichiometric quantities of high-purity raw materials: $Fe_{3}P$ (99.95%), $Co_{2}P$ (99.9%), P (99%), Si (99.5%), and Ni (99.99%). Initially, the raw material powders underwent meticulous mixing and grinding using a planetary ball-milling instrument (Across International PQ-N04, Livingston, NJ, USA) for 24 h. Stainless steel containers were utilized, maintaining a mass ratio of 5:1 of milling balls to powder. The resulting powder was shaped by uniaxial compaction onto a cylindrical die under a pressure of 200 MPa to form pellets. These pellets, with a thickness of about 3 mm, were subsequently sealed in quartz ampoules filled with argon gas (~26.6 kPa), with an external diameter of 12 mm. The ampoules underwent controlled heating, with the temperature gradually raised to 1000 °C over 24 h, followed by an additional 24 h period at 1100 °C to facilitate the desired reaction and sintering process. Upon the completion of the heating cycle, the ampoules were swiftly quenched in room-temperature water to induce the formation of the $Fe_{2}P$-type phase. The resultant quenched material was then meticulously ground into finer particles using an agate mortar, preparing it for subsequent analysis and characterization aimed at comprehensively understanding its properties.

A Smartlab Rigaku diffractometer employing Cu-Kα radiation was used for powder X-ray diffraction, operated at 10 kW using Bragg–Brentano geometry. The XRD patterns were fitted using the Rietveld method via FULLPROF software (version January 2023) [32] to confirm the phase content. The composition stoichiometry and surface topography of our samples were analyzed with the Phenom Pro-X Desktop SEM, at 10 and 15 kV acceleration voltage.

Curie temperature (T_C_) values were determined using a thermo-magneto-gravimetric Perkin-Elmer (Pyris Diamond TG/TDA, PerkinElmer, Waltham, MA, USA) instrument. Magnetic or structural transitions could be observed via the mass loss or gain using an external small magnet. The samples were heated from ambient temperature to 900 °C at a rate of 20 °C/min in an argon atmosphere. Magnetic measurements were caried out using a vibrating sample magnetometer (VSM), with a maximum field of 2 T. Anisotropy measurements were performed on epoxy-orientated samples using a two-component glue, cured in the presence of an external field of 2 T. Both magnetic and anisotropy measurements were performed at room temperature (RT).

## 3. Results and Discussion

### 3.1. Structural Characterization

**Figure 1:**
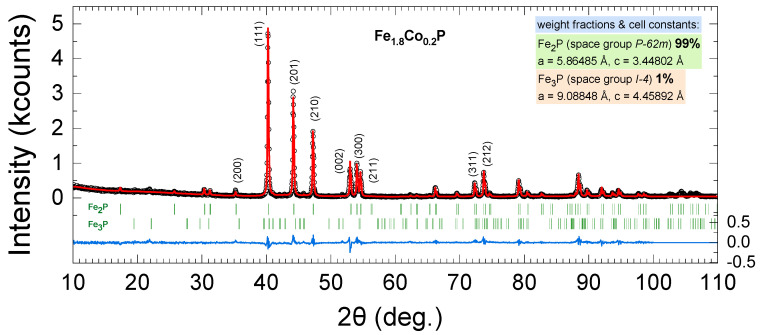
Rietveld plot of the compound $Fe_{1.8}Co_{0.2}P$.

Figure 1 presents the powder X-ray diffraction patterns of the $Fe_{1.8}{Co}_{0.2}P$ compound prepared by the ball milling of polycrystalline materials. The black dots illustrate the actual experimental data collected, while the red line depicts the predicted pattern based on the Rietveld method. Meanwhile, the blue line highlights the discrepancy between the experimental data and the Rietveld refinement. The Rietveld pattern is described using the T-C-H pseudo-Voigt function No. 7. The structural model that we used consists of the $Fe_{2}P$-type phase, which is characterized by space group No. 189, P-62m (Hermann–Mauguin symbol), in the hexagonal crystal system with a general multiplicity of 12, and the $Fe_{3}P$-type phase, described by space group No. 82, (I-4), in the tetragonal crystal system with a multiplicity of the general position of 8. The free parameters of the refinement include the cell constants and the Lorentzian isotropic strain and size parameters. The presence of sharp and distinct diffraction peaks in Figure 1 suggests that the sample possesses high crystallinity, indicating a well-ordered atomic structure. The weight fraction of the $Fe_{2}P$-type phase is 99%, suggesting that the sample is composed almost entirely of one phase, with no significant presence of other phases. The Rietveld analysis determined that the lattice constants of the $Fe_{2}P$-type phase are a=5.8648(1) Å and c=3.4480(1) Å, resulting in a cell volume of $102.71(1)\;{Å}^{3}$. Additionally, the isotropic strain parameter is X~0 and the isotropic size parameter is Y=0.06901(1), which yields an average apparent size of 822(1) Å. The refinement converged with a Bragg factor $R_{B}=9.40\%$ for the Fe_2_P–type phase.

**Figure 2:**
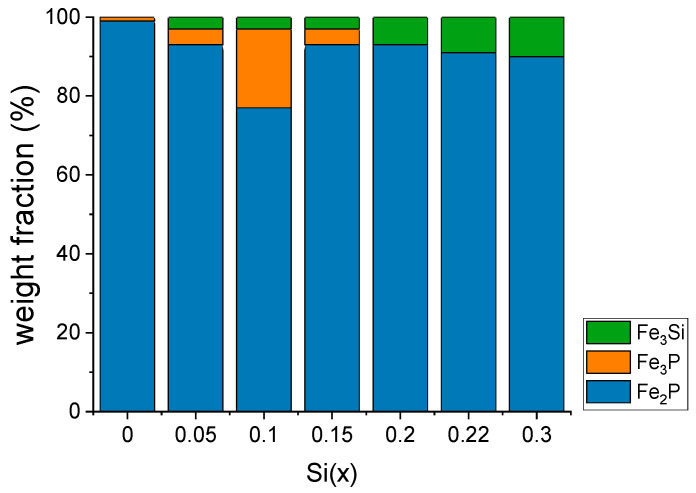
Weight fractions (%), versus Si(x). $Fe_{3}P$ and $Fe_{3}Si$ phases increase against the $Fe_{2}P$-type phase with increasing Si amount.

With the substitution of Si, the $Fe_{3}P$-type phase and the $Fe_{3}Si$ phase appear alongside the original $Fe_{2}P$-type phase [33,34]. According to a recent work [35], the addition of silicon to the compound has been found to extend the stability of the hexagonal phase; however, it is suspected to contribute to the formation of impurity phases. Consequently, the substitution of P with Si makes it difficult to form a single-phase material. Figure 2 displays the weight fractions (%) of the (Fe,Co)_2_P_1−x_Si_x_ compound against the amount of silicon. The Fe_3_P and Fe_3_Si phases increase relative to the Fe_2_P-type phase with increasing Si content.

**Figure 3:**
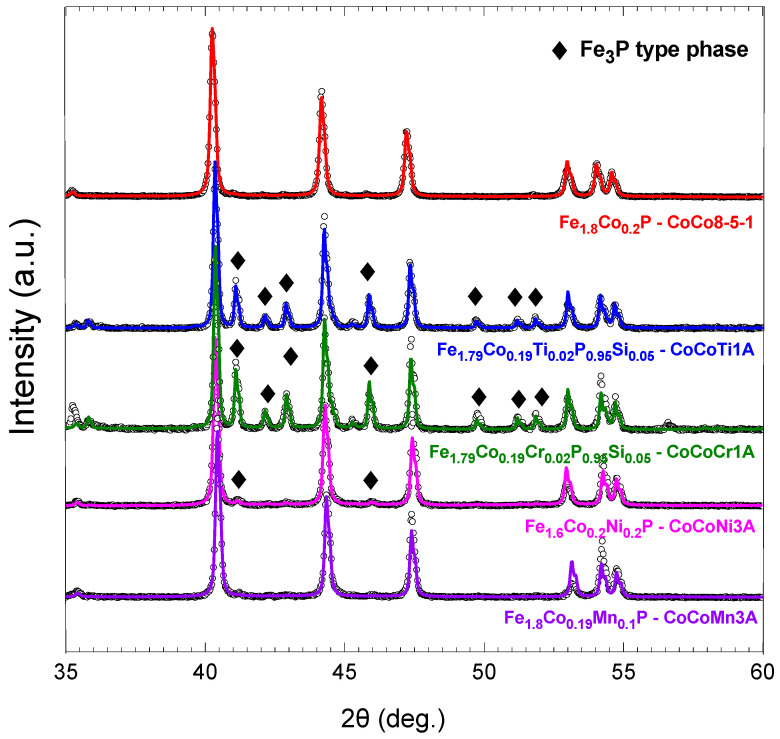
Rietveld plots of $Fe_{2-x-y}Co_{x}{\left(Ti,Cr,Ni,Mn\right)}_{y}(P_{z-1},{Si}_{z})$ samples. The Bragg peaks of the Fe_3_P-type phase are indicated by special diamond symbols.

**Figure 4:**
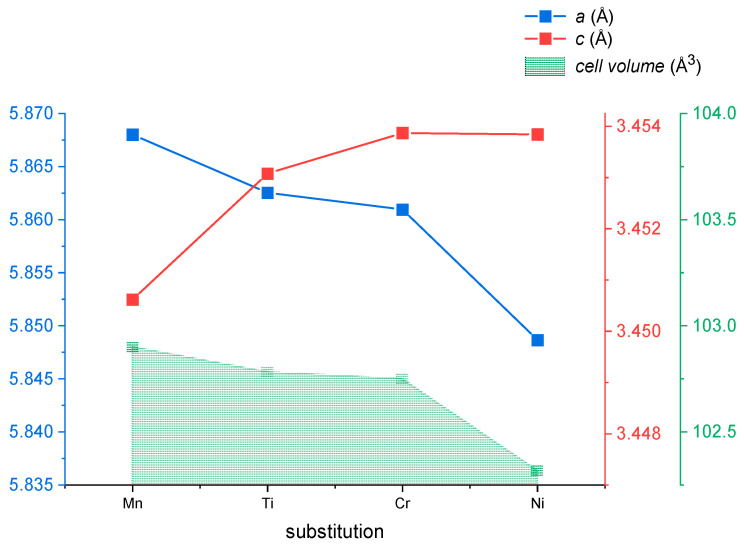
Cell constants of Fe_2_P-type phase vs. element substitution of $Fe_{2-x-y}Co_{x}{\left(Ti,Cr,Ni,Mn\right)}_{y}(P_{z-1},{Si}_{z})$.

Figure 3 shows the Rietveld plots of the sample with no additional element added (x=0.2, y=z=0), alongside samples with Ti, and Cr (x=0.2, y=0.02, z=0.05), Ni (x=0.2, y=0.2, z=0), and Mn (x=0.2, y=0.1, z=0) substitutions. The Bragg peaks of the Fe_3_P-type phase are indicated by special diamond symbols. The samples with Mn and Ni substitutions remain stable in the $Fe_{2}P$-type phase, while Ti and Cr substitutions lead to the formation of phase mixtures. The weight fraction of the $Fe_{2}P$-type phase is 74% for Ti and 68% for Cr, whereas Ni and Mn substitutions show higher proportions of 98% for Ni and 99% for Mn. The samples with Ti and Cr substitutions also include a small amount of Si in their composition, which complicates the formation of a single-phase material [35]. As element substitution is introduced, the lattice constants change, resulting in a subtle shift in the Bragg peaks to higher angles, as observed in the analysis. Table 1 shows the weight fraction (%) of the $Fe_{2}P$-type phase, along with the lattice constants (Å) and cell volume (${Å}^{3}$) of these samples, while Figure 4 visually illustrates how these values change with various substitutions. It is worth mentioning that the a and c lattice parameters change with various substitutions. More specifically, with substitutions of Mn, Ti, Cr, and Ni elements, the a lattice constant decreases while the c lattice constant increases. Notably, despite these opposing changes in the lattice constants, the overall cell volume remains constant, except for in Ni substitution, where it decreases.

### 3.2. Magnetic Characterization

**Figure 5:**
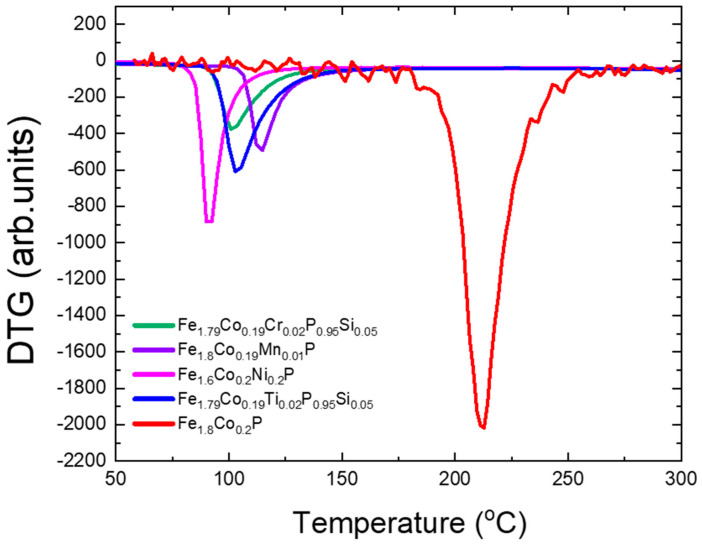
Thermogravimetric measurements of $Fe_{2-x-y}Co_{x}{\left(Ti,Cr,Ni,Mn\right)}_{y}(P_{z-1},{Si}_{z})$, to determine the T_C_ of the $Fe_{2}P$-type phase.

Thermogravimetric measurements of $Fe_{2-x-y}Co_{x}{\left(Ti,Cr,Ni,Mn\right)}_{y}(P_{z-1},{Si}_{z})$ are shown in Figure 5. The measurement of the pure stoichiometric sample $Fe_{1.8}Co_{0.2}P$ shows a Curie point at $212\left(5\right)$ °C, associated with the $Fe_{2}P$-type phase. This temperature marks a first-order magnetic phase transition from a ferromagnetic state to a paramagnetic one. This transition is thought to be linked to magnetoelastic phenomena, implying that the exchange energy is closely tied to how closely the atoms are packed together. The manner in which this transition unfolds is also sensitive to external factors such as the presence of impurities, deviations from the ideal stoichiometric mix, and the intensity of any external magnetic fields applied to the sample. The substitution of Ti, Cr, Ni, and Mn in the $Fe_{2-x-y}Co_{x}{\left(Ti,Cr,Ni,Mn\right)}_{y}(P_{z-1},{Si}_{z})$ samples leads to lower Curie points: Mn substitution results in the highest Curie point $117\left(2\right)$ °C, while Ni substitution leads to the lowest at $92\left(3\right)$ °C. Figure 5 shows the change in the Curie points versus element substitution.

**Figure 6:**
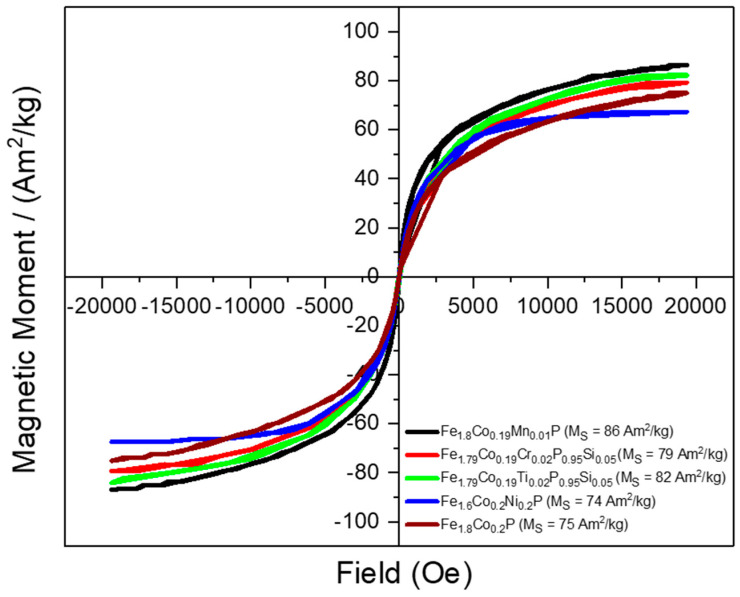
VSM magnetic measurements at RT of $Fe_{2-x-y}Co_{x}{\left(Ti,Cr,Ni,Mn\right)}_{y}\left(P_{z-1},{Si}_{z}\right)$ samples.

All samples were magnetically characterized at room temperature. The VSM data collected at room temperature are illustrated in Figure 6. The magnetization measurements reveal a general trend where substitutions lead to an increase in saturation magnetization, compared to the pure $Fe_{1.8}Co_{0.2}P$ sample or Ni substitution, which exhibits $M_{S}=75\left(1\right)\;\mathrm{and}\;74\left(1\right)Am^{2}/kg$, respectively. This reduction in $M_{S}$ with nickel indicates that the substituted elements affect the magnetic domains and their alignment, leading to a weaker collective magnetic field. However, Mn substitution yields the highest value of $M_{S}$: approximately $86(1)Am^{2}/kg$. This suggests that Mn has a unique influence on the magnetic properties of the sample, enhancing the alignment of magnetic domains and contributing to a stronger overall magnetic response. Z.Q. Ou et al. established with neutron diffraction that Mn preferentially substitutes the 3g site in Fe_2_P and carries a magnetic moment of 2.5–2.9 μ_B_/Mn, which is significantly larger than that of Fe [36]. Compared to this work, our system consists of a much smaller amount of Mn (y=0.1)  in the $Fe_{2-x-y}Co_{x}{\left(Ti,Cr,Ni,Mn\right)}_{y}(P_{z-1},{Si}_{z})$ compound. The specific electronic and atomic interactions introduced by Mn substitution warrant further investigation to fully understand their impact on the material’s magnetic behavior. Table 2 provides an overview of the Curie points and the saturation magnetization values for the $Fe_{2-x-y}Co_{x}{\left(Ti,Cr,Ni,Mn\right)}_{y}(P_{z-1},{Si}_{z})$ samples.

The microstructure of materials significantly affects their magnetic properties. For instance, in alloys like those studied in our work, variations in composition can lead to changes in crystal structure, grain size, and phase distribution. The relationship between cell constants and saturation magnetization is complex but significant. Changes in lattice constants due to substitution can modify interatomic distances, which affect exchange interactions among magnetic ions. These modifications may enhance or reduce $M_{S}\;$ depending on whether they strengthen or weaken ferromagnetic coupling within the material. Details of these effects are given in previous publications [35,37].

### 3.3. Ku Variation Versus Si

**Figure 7:**
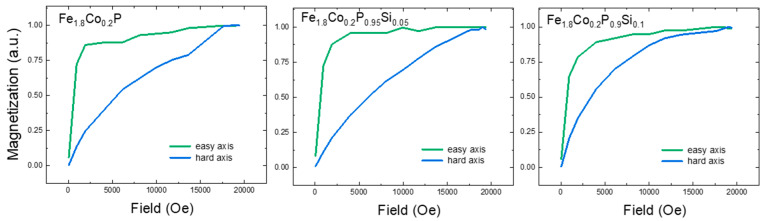
Anisotropy curves in orientated compound $Fe_{2-x}Co_{x}(P_{z-1},{Si}_{z})$, with x=0.2, z=0, 0.05, 0.1.

The $K_{u}$ anisotropy parameter quantifies the energy required to reorientate the magnetization of a material away from its easy axis. To determine the $K_{u}$, we first ground the $Fe_{2-x}Co_{x}(P_{z-1},{Si}_{z})$ samples into a fine powder. This powder was then evenly mixed with a two-part epoxy resin, aiming for a nearly equal proportion of sample to adhesive. The resulting blend was shaped into rectangular plates with dimensions of $10\times 10\times 1\;{mm}^{3}$. To finalize the process, these plates were placed at the core of a strong permanent magnet (1T), taking care to align the surface of the plates at a right angle to the magnetic field’s orientation with the sample axis, orientated along or perpendicular to the external field. The $K_{u}$ parameter is calculated by integrating the difference in effective magnetic fields along the two axes (the hard and the easy axis) over the range of magnetization from 0 to saturation. The expression is modified to account for the additional energy contribution from the demagnetizing field [38]:

(1) $$K_{u}=\int_{0}^{M_{S}}\left(H_{eff}^{a-axis}-H_{eff}^{c-axis}\right)dM,$$

where $H_{eff}^{a-axis}$ and $H_{eff}^{c-axis}$ are the effective magnetic fields along the a and c axis (the hard and the easy axis) of the material and $M_{S}$ is the saturation magnetization of the material. The calculation has been refined to include both the phase percentage and the normalization factor, ensuring a more precise and representative outcome. Table 3 shows the $K_{u}$ anisotropy parameter values versus the Si amount in the $Fe_{2-x}Co_{x}(P_{z-1},{Si}_{z})$ samples for z=0,0.05,0.1. The sample with z=0.05 has the highest value for the anisotropy constant $K_{u}$. Figure 7 displays the anisotropy curves obtained via VSM measurements in epoxy-orientated samples.

## 4. Conclusions

Substitutions in $Fe_{2}P$ alloys offer a promising avenue for enhancing their magnetic properties for permanent magnet applications. By carefully selecting and controlling substitutional elements, researchers can tailor the magnetic behavior of $Fe_{2}P$ alloys to meet the requirements of specific industrial applications such as rare-earth-free permanent magnets. Despite the progress made in understanding the effects of substitutions on the magnetic properties of $Fe_{2}P$ alloys, several challenges remain. These include the need for precise control over composition and microstructure, as well as the development of scalable synthesis methods. Based on the results of our work, the optimal combination for enhancing the magnetic properties of Fe_2_P alloys for permanent magnets involves a combined substitution of Fe with Co and P with Si, and fine tuning with other transition metals or metalloids, which is described by the formula [Fe_2−x_ (Co,Mn,Ni,Ti)_x_ (P_1−y−z_Si_y_B_z_)]. Future research directions may focus on computational modeling, advanced characterization techniques, and novel alloy design strategies to further optimize the magnetic performance of $Fe_{2}P$-based permanent magnets. A multielement substitution approach based on the concept of compositional complex alloys is underway to optimize the system’s properties toward “gap” magnet fabrication.

## Figures and Tables

**Table 1:** Comparison of the weight fraction (%) of the Fe_2_P-type phase, the lattice constants (Å), and the cell volume (${Å}^{3}$) between $\;Fe_{2-x-y}Co_{x}{\left(Ti,Cr,Ni,Mn\right)}_{y}(P_{z-1},{Si}_{z})$ samples.

Compound	$Fe_{2}P$-Type ph. w. fr. (%)	a(Å)	c(Å)	$Volume({Å}^{3})$
$Fe_{1.8}Co_{0.2}P$	99	5.8648(1)	3.4480(1)	102.71(1)
$Fe_{1.8}Co_{0.19}Mn_{0.01}P$	99	5.8680(1)	3.4506(1)	102.90(1)
$Fe_{1.79}Co_{0.19}Ti_{0.02}P_{0.95}Si_{0.05}$	74	5.8625(2)	3.4530(2)	102.78(1)
$Fe_{1.79}Co_{0.19}{Cr}_{0.02}P_{0.95}Si_{0.05}$	68	5.8609(2)	3.4538(2)	102.75(1)
$Fe_{1.6}Co_{0.2}Ni_{0.2}P$	98	5.8486(1)	3.4538(1)	102.32(1)

**Table 2:** Curie temperature $T_{C}({}^{\circ}C)$ for the $F_{2}P$-type phase and saturation magnetization $M_{S}(Am^{2}/kg)$ of $Fe_{2-x-y}Co_{x}{\left(Ti,Cr,Ni,Mn\right)}_{y}\left(P_{z-1},{Si}_{z}\right)$ samples.

Compound	$T_{C}({}^{\circ}C)$	$M_{S}(Am^{2}/kg)$
$Fe_{1.8}Co_{0.2}P$	212(5)	75(1)
$Fe_{1.8}Co_{0.19}Mn_{0.01}P$	117(2)	86(1)
$Fe_{1.79}Co_{0.19}Ti_{0.02}P_{0.95}Si_{0.05}$	105(3)	82(1)
$Fe_{1.79}Co_{0.19}{Cr}_{0.02}P_{0.95}Si_{0.05}$	100(3)	79(1)
$Fe_{1.6}Co_{0.2}Ni_{0.2}P$	92(3)	74(1)

**Table 3:** $K_{u}\;(MJ/m^{3}),$ anisotropy variation versus Si amount in the $Fe_{2-x}Co_{x}(P_{z-1},{Si}_{z})$ samples, with z=0, 0.05, 0.1.

Compound	$K_{u}\;(MJ/m^{3})$
$Fe_{1.8}Co_{0.2}P$	0.4327
$Fe_{1.8}Co_{0.2}P_{0.95}Si_{0.05}$	0.4882
$Fe_{1.8}Co_{0.2}P_{0.9}Si_{0.1}$	0.2891

## Data Availability

The original contributions presented in this study are included in the article. Further inquiries can be directed to the corresponding author.
